# An Advanced Multi-Sensor Acousto-Ultrasonic Structural Health Monitoring System: Development and Aerospace Demonstration

**DOI:** 10.3390/ma10070832

**Published:** 2017-07-20

**Authors:** Joel Smithard, Nik Rajic, Stephen van der Velden, Patrick Norman, Cedric Rosalie, Steve Galea, Hanfei Mei, Bin Lin, Victor Giurgiutiu

**Affiliations:** 1 Monash University, Clayton 3800, Australia; joel.smithard@dst.defence.gov.au; 2 Aerospace Division, Defence Science and Technology Group, 506 Lorimer St, Fishermans Bend 3207, Australia; Stephen.VanderVelden@dst.defence.gov.au (S.v.d.V.); Cedric.Rosalie@dst.defence.gov.au (C.R.); Steve.Galea@dst.defence.gov.au (S.G.); 3 QineteQ Australia, 506 Lorimer St, Port Melbourne 3207, Australia; Patrick.Norman@dst.defence.gov.au; 4 Laboratory for Active Materials and Smart Structures, University of South Carolina, Columbia, SC 29208, USA; Hanfei.Mei@cec.sc.edu (H.M.); LINBIN@cec.sc.edu (B.L.); VICTORG@sc.edu (V.G.)

**Keywords:** structural health monitoring, acousto-ultrasonics, Piezoelectric Wafer Active Sensor, Fiber Bragg Grating, electromechanical impedance, instrumentation, aerospace composite materials

## Abstract

A key longstanding objective of the Structural Health Monitoring (SHM) research community is to enable the embedment of SHM systems in high value assets like aircraft to provide on-demand damage detection and evaluation. As against traditional non-destructive inspection hardware, embedded SHM systems must be compact, lightweight, low-power and sufficiently robust to survive exposure to severe in-flight operating conditions. Typical Commercial-Off-The-Shelf (COTS) systems can be bulky, costly and are often inflexible in their configuration and/or scalability, which militates against in-service deployment. Advances in electronics have resulted in ever smaller, cheaper and more reliable components that facilitate the development of compact and robust embedded SHM systems, including for Acousto-Ultrasonics (AU), a guided plate-wave inspection modality that has attracted strong interest due mainly to its capacity to furnish wide-area diagnostic coverage with a relatively low sensor density. This article provides a detailed description of the development, testing and demonstration of a new AU interrogation system called the Acousto Ultrasonic Structural health monitoring Array Module^+^ (AUSAM^+^). This system provides independent actuation and sensing on four Piezoelectric Wafer Active Sensor (PWAS) elements with further sensing on four Positive Intrinsic Negative (PIN) photodiodes for intensity-based interrogation of Fiber Bragg Gratings (FBG). The paper details the development of a novel piezoelectric excitation amplifier, which, in conjunction with flexible acquisition-system architecture, seamlessly provides electromechanical impedance spectroscopy for PWAS diagnostics over the full instrument bandwidth of 50 KHz–5 MHz. The AUSAM^+^ functionality is accessed via a simple hardware object providing a myriad of custom software interfaces that can be adapted to suit the specific requirements of each individual application.

## 1. Introduction

Embedded structural health monitoring (SHM) systems offer the prospect of enabling inspection for structural damage on-demand. For the aircraft industry, this represents a significant opportunity to improve on traditional maintenance practice which relies on a prescriptive regime of manual non-destructive inspection (NDI), a labor intensive and time consuming process that also often involves extensive structural disassembly for access to critical areas within an airframe.

Acousto-ultrasonics (AU) is a guided plate-wave based inspection modality that offers wide-area diagnostic coverage with a relatively low sensor density and can be applied to both metallic and composite structures [1,2,3]. The method typically relies on surface mounted or embedded Piezoelectric Wafer Active Sensor (PWAS) elements to generate the interrogating wave field and can employ either PWAS elements or optical Fiber Bragg Gratings (FBG) [4] to sense the scattered field. Interest in optical fiber sensing of plate-waves has grown considerably in recent years, driven in part by concerns that piezoelectric materials may not operate reliably under severe mechanical loading over an extended service life [5]. Optical fiber sensors are not only more durable than piezo-ceramic sensors but are also immune to electromagnetic interference, have a small footprint which allows them to be easily embedded in fiber composites, and in the case of FBGs are readily multiplexed allowing for distributed sensing. Recent studies have shown that FBG sensors can achieve high bandwidths and are thus capable of detecting higher order Lamb waves [6] and when written in a high-density array are capable of resolving multi-modal wave packets into constituent modes [7,8,9].

Irrespective of the type of sensor employed, a key requirement for the successful transition of AU into operational use on aircraft and other high value engineering assets is an instrumentation platform that is easy to use, compact, portable, low in mass and, electrically and mechanically robust. Highly flexible and inexpensive instrumentation for basic laboratory investigations is also required to allow researchers to continue working on the various scientific and engineering issues that are impeding industry transition. Traditional Commercial-Off-The-Shelf (COTS) systems were bulky, costly and often inflexible in configuration and/or scalability, militating against in-service deployment. With ever increasing access to smaller and more energy efficient electronics newer generation hardware has gone a long way to solving these issues [10,11,12,13,14]. However, there is still considerable room for improvement, particularly with respect to drive voltage, excitation and acquisition bandwidth, system compactness, portability and, electrical and mechanical robustness.

This paper reports on the development of a light-weight, robust, compact, portable and inexpensive device for AU excitation and interrogation called the Acousto Ultrasonic Structural health monitoring Array Module^+^ (AUSAM^+^). The AUSAM^+^ has the footprint of a typical smart phone, provides autonomous control of four send and receive PWAS elements which can be operated in pitch-catch or pulse-echo regimes and can undertake electro-mechanical (EM) impedance measurements for transducer and structural diagnostics. The module also caters for fiber optic sensing of acoustic waves with four intensity-based Positive Intrinsic Negative (PIN) photodiodes, and can additionally acquire temperature and strain measurements. The development of both a Matlab and Python hardware object enables straightforward access to the full functionality of the device in both languages and thereby provides enormous flexibility for the creation of custom interfaces. This article describes the system from its conceptual foundation through to its design and development. The efficacy of the system is demonstrated through the results of first-of-class testing, as well as multiple laboratory AU studies on aerospace related metallic and composite structures using an array of PWAS elements and FBG sensors.

## 2. AUSAM^+^ Design and Methodology

### 2.1. Overview

The AUSAM^+^ was developed largely in response to performance limitations identified in its predecessor the AUSAM [2]. One of the primary performance-related design goals for the new device was an extended bandwidth to allow for the excitation of higher-order AU modes. Such excitations require high drive voltages on the order of 200 Vp-p across PWAS elements with bulk capacitance values of 1–10 nF. This initiated a major redesign of the High Voltage Drive Amplifier (HVDA) and acquisition system. A self-diagnostic capability was also prescribed to enable the structural integrity of the PWAS elements and their bond to the host structure to be assessed on demand. This required the incorporation of an EM impedance measurement capability. Given the rapidly growing interest in FBG sensing of plate waves, an ability to interface with these sensors was also incorporated in the design of the new system.

**Figure 1:**
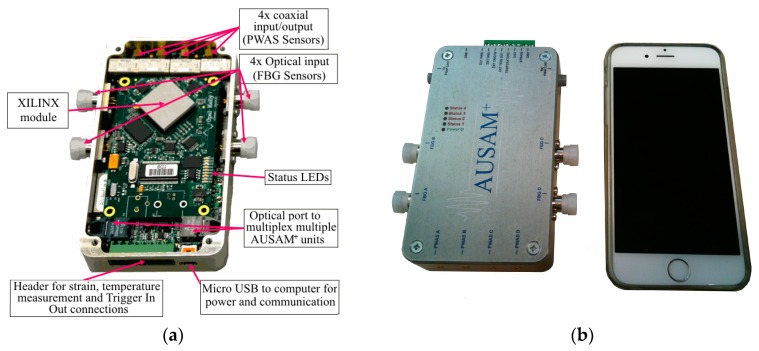
Acousto Ultrasonic Structural health monitoring Array Module^+^ (AUSAM^+^) system: (**a**) Inputs and Outputs; (**b**) Size comparison to an iPhone 6.

A fixed 50 MHz sample rate for both the acquisition system and HVDA achieved the target 50 kHz–5 MHz AU bandwidth while simplifying the anti-aliasing filter design. A commercial Field Programmable Gate Array (FPGA) module was included for high speed deserialization that also provided the required memory, ancillary computation and USB 2.0 compliant interface for power and communications. The remaining AUSAM^+^ hardware comprises several relatively distinct circuit sub-systems, all carefully separated spatially and electrically through the judicious use of ground and power planes on a single 6-layer Printed Circuit Board (PCB). This approach provides good HVDA cross-talk immunity whilst simultaneously maximizing noise-floor performance in a compact 115 × 65 × 20 mm^3^ aluminum enclosure (see Figure 1).

**Figure 2:**
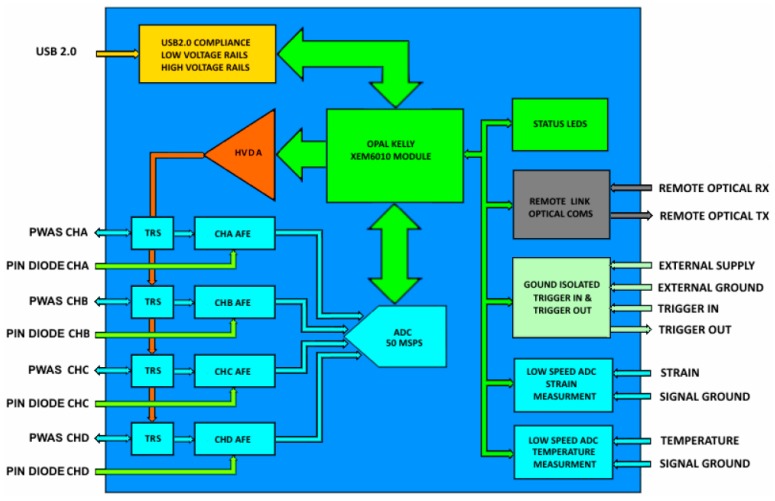
AUSAM^+^ simplified block diagram.

The configurable AUSAM^+^ acquisition system supports four low noise independent channels, labelled A through D, each with +55 dB of programmable gain. AU data can be acquired from either PWAS or PIN photodiodes for FBG sensor measurement and digitized in the high-speed Analog to Digital Converter (ADC); see simplified block diagram in Figure 2. One special configuration of the acquisition system allows simultaneous monitoring of the excitation voltage and current waveforms, providing invaluable information about the excitation quality and the electromechanical state of a connected PWAS. Monitoring the excitation waveforms also provides enormous scope for iterative algorithms to fine-tune the HVDA waveform generation. An integrated Transmit Receive Switch (TRS) on each channel allows excitation and acquisition to coexist seamlessly for both pulse-echo and pitch-catch interrogation regimes.

**Figure 3:**
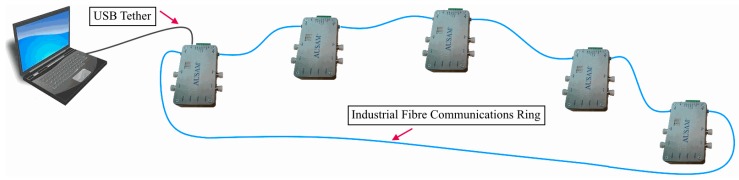
Industrial Fiber Communication Ring for channel expansion and scalability. Up to 62 AUSAM^+^ can exist on the Industrial Fiber Communications Ring (IFCR).

Variations in operational loads and temperatures can often impact on the acoustic response of a structure so to ensure these effects can be remedied, low-speed ADC circuitry was incorporated to support the measurement of temperature and strain. Further ancillary resources include ground isolated triggers and an Industrial Fiber Communications Ring (IFCR) for remote control of the module. The noise immune IFCR enables synchronized expansion of channels by adding up to 62 AUSAM^+^ together, thus providing an easily scalable SHM capability as illustrated in Figure 3. When multiple AUSAM^+^ are controlled over the IFCR, power is typically supplied to remote units by battery for improved noise performance, or an external power supply if noise is not an issue. All this functionality is managed by an intuitive hardware object that can be driven by a custom Graphical User Interface (GUI) or script written in either Matlab or Python.

### 2.2. Acquisition System Architecture

**Figure 4:**
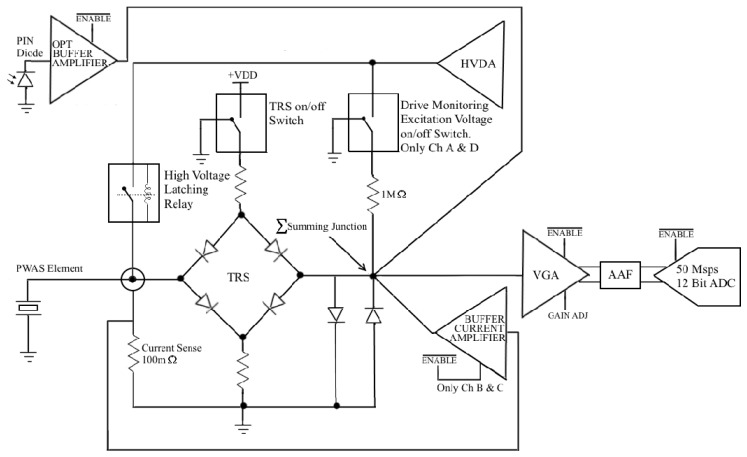
AUSAM^+^ simplified Analogue Front End (AFE) channel architecture, where the four available channels are referenced A through D. The Drive Voltage Monitoring on/off switch and 1 MΩ divider resistor only exists on channel A and channel D while the Buffer Current Amplifiers, of which there are only two, have their outputs connected to channel B and channel C only.

The four PWAS connectors and four PIN photodiodes are routed to four independent Analogue Front End (AFE) channels before being digitized in the four-channel high speed 12 bit 50 Msps ADC. Each AFE channel, represented by the simplified drawing in Figure 4, comprises a TRS, programmable +55 dB Variable Gain Amplifier (VGA), Anti-Aliasing filter (AAF) and various amplifier buffers and configurable switches.

These configurable AFE channels are pivotal in providing the AUSAM^+^ flexibility. Each channel TRS can be completely disabled to isolate any connected PWAS allowing the AFE channel to measure the PIN photodiode response or to monitor PWAS excitation voltage and current. These multiple sources, available at an AFE channel’s summing junction, are software selected with appropriate enable signals and switch configurations. Finally, each PWAS can be independently connected to the HVDA by a latching relay for actuation.

The special configuration applied to the four AFE channels that allow simultaneous monitoring of PWAS excitation voltage and current is called Drive Monitoring. When this mode is selected, AFE channel configurations are applied automatically via software based on which PWAS is presently connected to the HVDA. In order to accommodate simultaneous monitoring of excitation voltage and current on any individually selected PWAS, two AFE channels retain the option to measure excitation voltage and the remaining two are equipped to measure excitation current. How each of the four AFE channels A through D is configured during this mode is listed in Table 1.

As this AFE circuitry is particularly sensitive to noise it’s located on the bottom of the PCB using its own low noise ground plane. To further improve noise performance, the active sensing of the AUSAM^+^ occurs while noisy supplies are switched off and power rails remain temporarily energized by super capacitors. Two of the low speed ADC’s provide the ancillary strain gauge and temperature measurement while the third allows static optical power measurements on the user selected PIN photodiode. This static optical power measurement is extremely useful to diagnose the correct operation of the optical system when interrogating FBG sensors.

### 2.3. High Voltage Drive Amplifier Architecture

**Figure 5:**
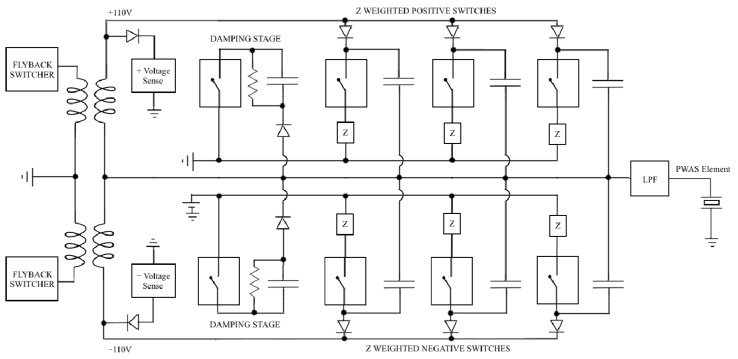
AUSAM^+^ simplified High Voltage Drive Amplifier (HVDA) (not all swich elements shown).

The novel HVDA architecture used to excite PWAS elements over the full AUSAM^+^ bandwidth sits on top of the PCB as far as possible from the noise sensitive AFE channels along with the bulk of the switched and linear power supplies. Exciting high frequencies up to 200 Vp-p over elements with bulk capacitances ranging from 1 to 10 nf demands a HVDA that can deliver large currents yet be flexible enough to iteratively adapt to reactive loads. The HVDA circuit achieves this by hybridizing a discrete bipolar digital-to-analogue converter with some class D amplifier architecture, illustrated in the simplified diagram of Figure 5.

Each HVDA switch element is associated with its own capacitor that can be charged simultaneously with all others up to a maximum of ±110 V respectively. Charging these capacitors from low internal voltages is performed by switching high efficiency fly-back transformers through steering diodes. Once all switch element capacitors are charged they collectively produce two large capacitor banks with opposite polarity while internal comparators in the sense circuitry provide feedback to regulate this voltage.

Each HVDA capacitor bank is approximately three orders of magnitude larger than the typical PWAS bulk capacitances intended to be driven by the AUSAM^+^ and once charged provide significant authority over the PWAS. Momentarily connecting a portion of either polarity HVDA capacitor bank in parallel with the connected PWAS through appropriately activated switch elements causes charge to flow into or out of the PWAS respectively. Thus, with judicious control of the many weighted switching elements one can generate an arbitrary PWAS voltage waveform.

The HVDA does not readily run out of charge under repetitious workloads due to the relatively small PWAS bulk capacitances, high excitation frequencies and small cycle numbers involved in typical AU windowed drive signals. In fact the power dissipated is marginal under heavy workloads compared to that required to run the acquisition system allowing the AUSAM^+^ to remain under the USB 2.0 maximum power specification of 2.5 W.

The first switch element damping stage provides a low impedance path to ground for the connected PWAS at critical times. This not only ensures that no inadvertent acoustic energy is produced in the PWAS while the amplifier capacitor banks are charging, but can also be used to momentarily damp the PWAS energy at any time. This is particularly useful if exciting a PWAS close to resonance in pulse-echo mode, enabling the swift reduction of ringing immediately after excitation.

The combination of HVDA and TRS architecture produces a +1.65 V Direct Current (DC) signal over any connected PWAS elements and must be considered when grounding a specimen. This should have a negligible effect in most SHM situations and is therefore a minor trade-off for improved flexibility.

### 2.4. EM Impedance Measurement

A reliable sensor platform is a key pre-requisite for any SHM capability as repair or replacement of defective components may be difficult or costly particularly in the case of embedment. Failures due to de-poling, disbonding or fracture are possible when a PWAS element is exposed to severe mechanical loading [5] so a capacity to assess sensor system health is vital to ensuring confidence in SHM system outputs. The AUSAM^+^ addresses this issue by leveraging the acquisition systems Drive Monitor mode which simultaneously measures PWAS excitation voltage and current waveforms. Characterization of the acquisition system was performed by a Solatron 1260 impedance analyzer to make measurements of both VGA input impedance and relevant AFE transfer functions (TF). The measurements described in this section were performed on specific portions of the AFE channel signal chain when configured in Drive Monitor mode and subsequently used to calibrate the EM impedance calculation.

#### 2.4.1. Excitation Voltage Measurement

**Figure 6:**
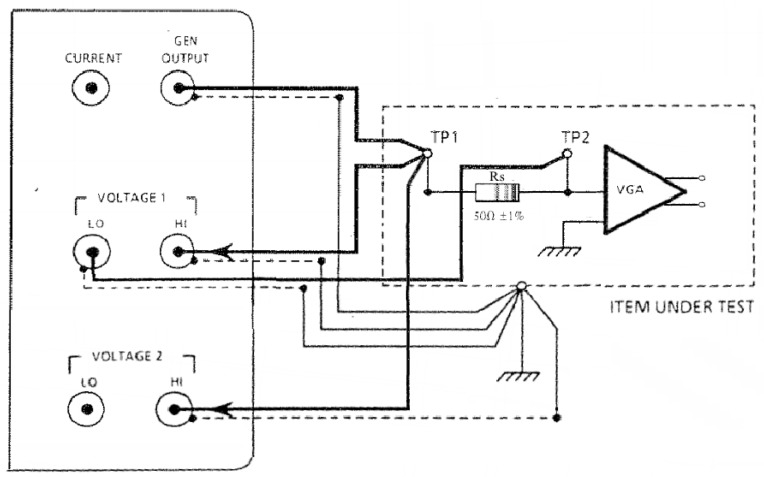
Solatron 1260 measurement of the Variable Gain Amplifier (VGA) input impedance when the AFE channel is configured to monitor PWAS excitation voltage in Drive Monitor mode. The impedance between TP1 and ground is$\;Z_{T-\mathrm{voltage}}\left(\omega \right)$. The impedance between TP2, which is located at the AFE channels summing junction, and circuit ground, is the VGA input impedance$\;Z_{I-\mathrm{voltage}}\left(\omega \right)$.

The VGA input impedance of an AFE channel, when configured for PWAS excitation voltage monitoring, was measured using a precision 50 Ω resistor shown in Figure 6. The Solatron generator output provided a logarithmic 250 point per decade sweep over the instrument bandwidth of 50 kHz to 5 MHz while providing measurements on its two Voltage amplifiers. Voltage 1 amplifier was configured for differential input and used to indirectly measure the current through the known resistance Rs. Voltage 2 amplifier, configured for single ended input with a grounded screen, measured the total applied generator output voltage.

(1) $$Z_{T-\mathrm{voltage}}\left(\omega \right)=\frac{{\mathrm{Voltage}}_{2}\left(\omega \right)}{\left[\frac{{\mathrm{Voltage}}_{1}\left(\omega \right)}{\mathrm{Rs}}\right]}=\mathrm{Rs}+Z_{I-\mathrm{voltage}}\left(\omega \right)$$

where $Z_{T-\mathrm{voltage}}\left(\omega \right)\;$is the total circuit impedance and, $Z_{I-\mathrm{voltage}}\left(\omega \right)\;$is the input impedance of the VGA when the AFE channel is configured for PWAS excitation voltage monitoring, viz.

(2) $$Z_{I-\mathrm{voltage}}\left(\omega \right)=\frac{{\mathrm{Voltage}}_{2}\left(\omega \right)}{\left[\frac{{\mathrm{Voltage}}_{1}\left(\omega \right)}{\mathrm{Rs}}\right]}-\mathrm{Rs}$$

**Figure 7:**
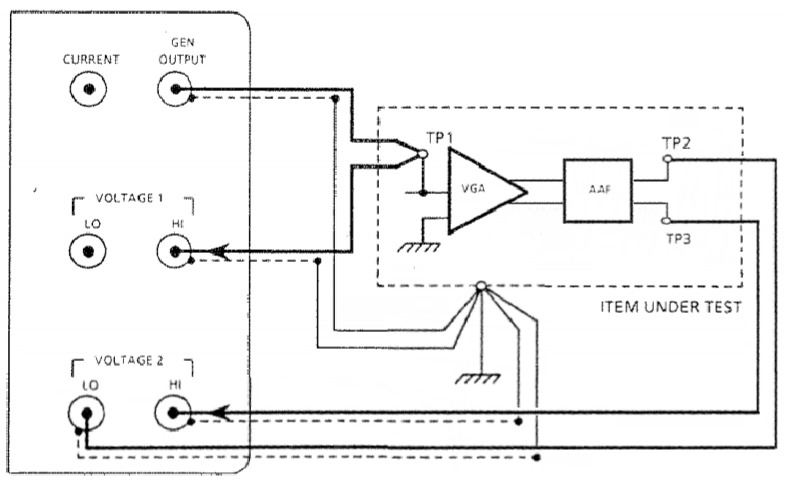
Solatron 1260 measurement of the transfer functions (TF) as the measured Piezoelectric Wafer Active Sensor (PWAS) excitation voltage signal propagates through the remaining AFE channel when configured in Drive Monitor mode. TP1 is located at the AFE channels summing junction while TP2 and TP3 comprise the differential signal$\;V_{ADC}\left(\omega \right)$ subsequently digitized by the high-speed Analog to Digital Converter (ADC).

The TF of the PWAS excitation voltage through the remaining AFE signal chain, comprising VGA and AAF, was also measured by the Solatron 1260 impedance analyzer in the configuration shown in Figure 7. This time Voltage 1 amplifier was configured for single ended input with grounded screens and measured the input to the signal chain while Voltage 2 amplifier was configured for differential input and measured the output of the signal chain.

This PWAS excitation voltage TF measurement $TF_{Voltage}\left(\omega \right)$ in addition to the VGA input impedance $Z_{I-\mathrm{voltage}}\left(\omega \right)$ calculated prior provides the necessary information to obtain the complete TF for the AUSAM^+^, $\mathrm{called}\;TF_{AUSAM-Voltage}\left(\omega \right)$ which is used to calibrate the PWAS excitation voltage measurement in Drive Monitor mode.

(3) $$TF_{AUSAM-Voltage}\left(\omega \right)=\frac{V_{ADC}\left(\omega \right)}{V_{EXC}\left(\omega \right)}=\left\{\frac{V_{EXC}\left(\omega \right).Z_{I-\mathrm{voltage}}\left(\omega \right)}{Z_{I-\mathrm{voltage}}\left(\omega \right)+1M\Omega }.TF_{Voltage}\left(\omega \right)\right\}÷V_{EXC}\left(\omega \right)\;TF_{AUSAM-Voltage}\left(\omega \right)=\frac{Z_{I-\mathrm{voltage}}\left(\omega \right)}{Z_{I-\mathrm{voltage}}\left(\omega \right)+1M\Omega }.TF_{Voltage}\left(\omega \right)$$

where $V_{ADC}\left(\omega \right)\;$is the voltage measured at the input to the high speed ADC and $V_{EXC}\left(\omega \right)\;$is the PWAS excitation voltage.

The 1 MΩ resistor appears in Equation (3) since the PWAS excitation voltage $V_{EXC}\left(\omega \right)$ is first divided down by a 1 MΩ resistor, recall Figure 4, before reaching the channels summing junction, which is located at the VGA input. The presence of the 1 MΩ resistor during the previous measurement of VGA input impedance $Z_{I-\mathrm{voltage}}\left(\omega \right)$ was ignored due to the 50 Ω resistor Rs dominating the source impedance of the VGA.

#### 2.4.2. Excitation Current Measurement

**Figure 8:**
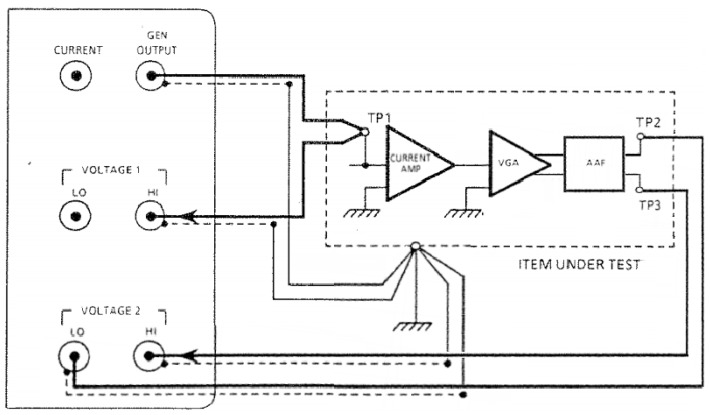
Solatron 1260 measurement of the TF as the measured PWAS excitation current signal propagates through the AFE channel when configured in Drive Monitor mode. TP1 correlates to the input of the current amplifier while TP2 and TP3 comprise the differential signal$\;V_{ADC}\left(\omega \right)$ subsequently digitized by the high-speed ADC.

There was no requirement to calculate the current amplifier input impedance since the source impedance, created by a 100 mΩ current sense resistor is so small in comparison, again recall Figure 4. However, since measuring the PWAS excitation current in Drive Monitoring mode uses an AFE signal chain configured differently to when measuring PWAS excitation voltage, the Solatron 1260 impedance analyzer was used to make a further TF measurement in the same manner as above with the 100 mΩ current sense resistor removed, see Figure 8. The TF of the PWAS excitation current measurement propagating through the AFE signal chain is$\;TF_{AUSAM-Current}\left(\omega \right)$.

#### 2.4.3. EM Impedance Calculation

The complex EM impedance measurement $Z_{\omega }$ of a connected PWAS element is calculated using a swept pulse spectroscopy approach. The pulses delivered are Hanning windowed functions stepped through a prescribed frequency range. Sampling each excitation pulse in Drive Monitor mode provides the voltage signal $V_{n}$ and current signal $I_{n}$ where n represents the sample number. With the application of corrections using $TF_{AUSAM-Voltage}\left(\omega \right)$ and $TF_{AUSAM-Current}\left(\omega \right)$ respectively, the subsequent Discrete Fourier Transform (DFT) of both series is calculated as follows;

(4) $$V_{k}=\sum_{n=0}^{N-1}I_{v}\cdot e^{-2\pi kn/N}$$

and,

(5) $$I_{k}=\sum_{n=0}^{N-1}I_{n}\cdot e^{-2\pi kn/N}$$

The DFT creates a sequence of complex coefficients $V_{k}$ and $I_{k}$ in the frequency domain where the complex impedance $Z_{k}$ at each frequency k is found by taking the ratio of the coefficients, viz,

(6) $$Z_{k}=\frac{V_{k}}{I_{k}}$$

of interest is only the corresponding frequency bin of $Z_{k}$ that matches the excitation frequency ω and provides the single complex pair that forms $Z_{\omega }$ at ω. Successive iterations of the process, for each new PWAS excitation frequency, provide more complex pairs that populate the full sweep of the EM impedance$\;Z_{\omega }$.

### 2.5. Firmware and Software Architecture

The AUSAM^+^ firmware is a compiled binary file that resides in the on-board flash non-volatile memory and configures the FPGA upon power up. Object oriented software, written in both Matlab and Python, provide the mechanism to control the AUSAM^+^ hardware by communicating to firmware over USB 2.0.

**Figure 9:**
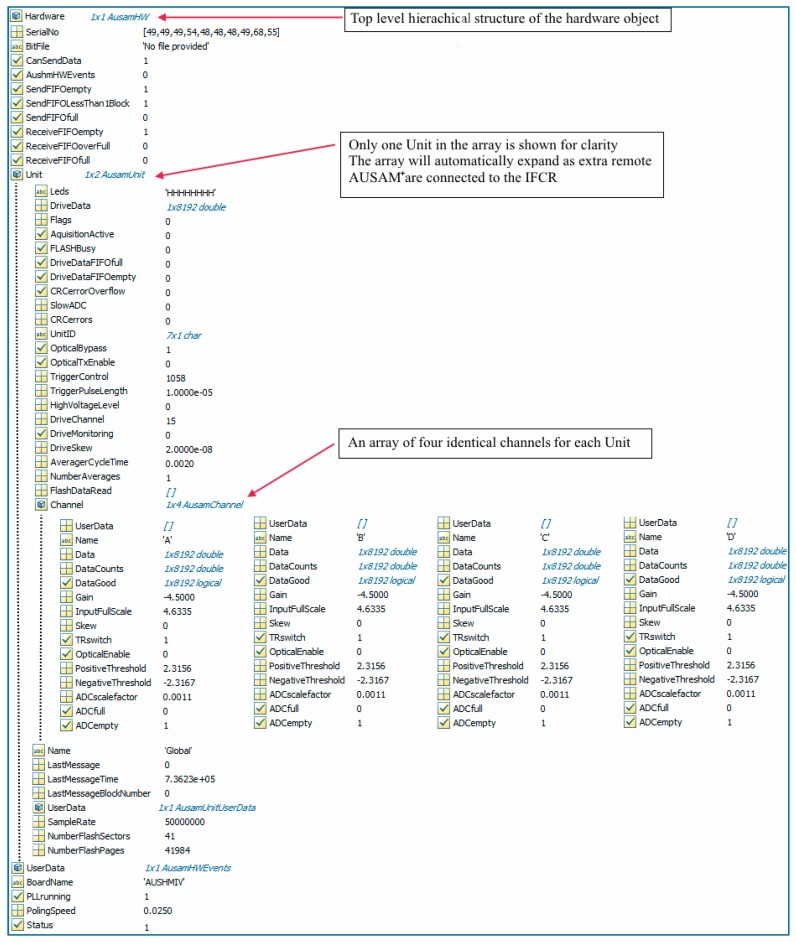
AUSAM^+^ hardware object hierarchy and contents.

All digital signal processing and other custom high level algorithmic functionality is handled by Matlab or Python code written around the hardware object, but future versions of the AUSAM^+^ will house a more powerful Xilinx FPGA chip running an Advanced RISC Machine (ARM) core processor on-board for complete autonomy. The hardware object holds all the attributes and settings to mirror the state of the physical hardware at any point in time. The hierarchy of the hardware object follows an intuitive design to allow rapid GUI or script development, as shown in Figure 9.

The hardware object’s first level holds information that is generic to all AUSAM^+^ connected either locally or remotely. The second level in the object hierarchy is the Unit. This array equals the quantity of AUSAM^+^ connected plus 1 on the IFCR. The first Unit object in the array is a global one, and if anything is altered in this global Unit then all AUSAM^+^ connected locally or remotely will be changed accordingly. The second Unit in the array is always the closest to the host PC and is tethered by the USB cable. Any further Units in the array are identified as remote AUSAM^+^ residing on the IFCR. The third level of the hardware object is the Channel. Each Unit has an array of 4 identical Channels as per the acquisition system. The information in the Channels corresponds to anything that is relevant to an individual AUSAM^+^ AFE channel configuration, including previously acquired ADC data etc. This simple hardware object architecture is both intuitive and scalable to meet the challenges of embedded SHM and rapid laboratory research.

**Figure 10:**
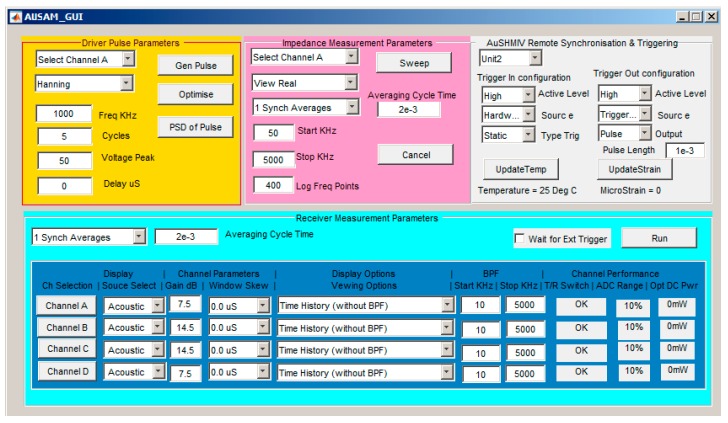
Example Graphical User Interface (GUI) created to explore the AUSAM^+^ capability.

Figure 10 is an example GUI created in Matlab showing some of the accessible features of the AUSAM^+^, however in practice, SHM tasks in the laboratory and beyond are typically implemented by rapid custom scripting utilizing the intuitive hardware object.

A remote interface unit has been tested to run the Python hardware object on a Linux operating system installed on a Raspberry Pi Zero System-on-Chip (SOC) computer. The remote interface provides options for USB Wi-Fi or 3G dongles opening up further flexibility for remote embedded SHM work. Currently, control of the Python hardware object on a Raspberry Pi Zero is over Wi-Fi through the use of Python remote procedure-call software. However all this functionality can be housed within the existing module by upgrading the Xilinx FPGA as mentioned previously.

## 3. AUSAM^+^ First of Class Assessment

### 3.1. Overview

Several main areas were highlighted for a First of Class Test (FOCT) including the HVDA, acquisition system, EM impedance measurement, and user functionality [15]. Not all of the planned FOCTs have been completed but sufficient work was done to establish confidence in the functionality and performance of the system as well as understand system limitations.

### 3.2. HVDA performace

**Figure 11:**
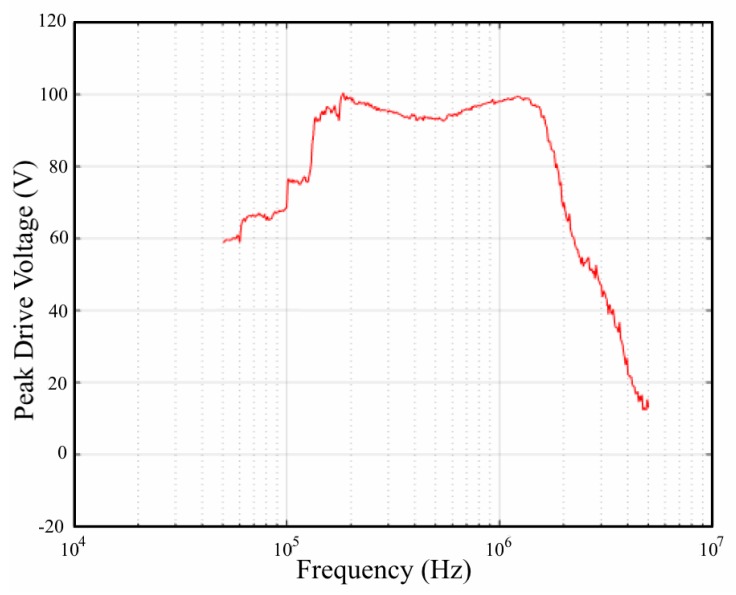
Typical variation in maximum excitation peak voltage with various excitation frequencies applied over a precision 1 nF capacitor.

The initial activity was to investigate the nominal HVDA performance, viz, actual excitation-signal power, quality and, frequency resolution. In this case the HVDA was swept through the frequency band of interest using a five cycle Hanning modulated sinusoidal signal at maximum amplitude applied to a 1 nF precision capacitor. The 1 nF capacitor was used to simulate the capacitance of a typical PWAS element without the influence of resonances. The final optimized excitation signal measured across the capacitor was compared to the HVDA desired output, both in the time and frequency domain; thereby measuring the excitation quality. The cross correlation coefficient between the desired output and final excitation signal indicated a value no less than 0.95 across the band of interest, while the frequency selectivity showed better than 1 kHz resolution. The peak drive voltage of the excitation signal over the 1 nF capacitor for the frequency range 50 kHz to 5 MHz is shown in Figure 11. It is believed that the peak excitation signal can be improved if a more realistic HVDA switch model is used in the algorithm to generate the initial excitation signal.

### 3.3. Acquisition System Performance

The next phase of the testing was to determine the performance of the acquisition system, viz, determine the Root Mean Square (RMS) noise-floor performance, channel cross-talk during excitation, and measurement accuracy. In these tests, the AUSAM^+^ measurements were compared with those taken using a 12 Bit LeCroy digital oscilloscope. The comparisons were taken in both Drive Monitoring mode and in the active acquisition regimes.

#### 3.3.1. AFE Cross-Talk and Noise Floor

**Figure 12:**
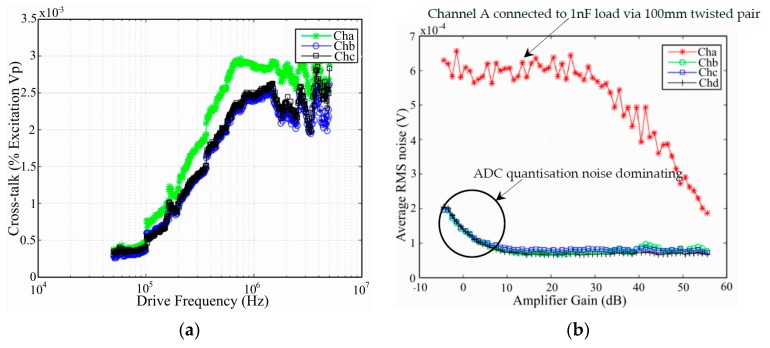
(**a**) Percentage of channel D excitation cross-talk on other channels; (**b**) Typical single-shot (no synchronous averaging) Root Mean Square (RMS) noise floor performance.

The channel cross-talk is frequency dependent so a sweep across the entire instrument bandwidth was performed with excitation voltage set to maximum. The data on each channel, excluding the drive channel, was collected and the peak cross-talk voltage recorded. Figure 12a shows the percentage of the excitation amplitude, when applied over a 1 nF precision capacitor attached to channel D, which couples into adjacent channels. The cross-talk plots were performed while the other channels were unterminated to avoid influences from connected loads. These results show cross-talk below 0.003% between the drive and the other channels at the most susceptible excitation frequencies.

The RMS noise floor was measured on each channel for configurations where one channel was connected to a 1 nF precision capacitor using 100 mm long twisted pair wire, while other channels were left open circuit with no load attached. The time history responses were recorded on each channel and any DC component removed before the RMS value was calculated. Typical average Vrms levels for increasing gain in each AFE channels VGA are shown in Figure 12b. The average RMS noise floor values calculated for channels un-attached to a load were less than 100 μVrms while drive channels that were coupled internally to the HVDA and externally to the 1 nF precision capacitor showed a larger figure, of not more than 700 μVrms (see channel A in Figure 12b). Note that in assessing the RMS noise floor the low gain region was not considered due to ADC quantization noise dominating the measurement. The PIN photodiode channels were tested for dark current noise and produced a noise floor of less than 300 μVrms.

#### 3.3.2. Measurement Accuracy

To assess the acquisition system measurement accuracy of the device, two scenarios were investigated. In both cases the AUSAM^+^ measurements were compared to those from a LeCroy oscilloscope which was band-limited to 20 MHz providing a similar roll off to the AUSAM^+^ AAF.

**Figure 13:**
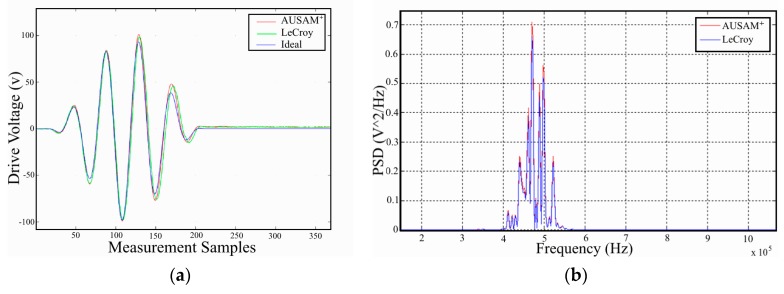
Acquisition system comparison to a LeCroy oscilloscope in both Drive Monitor mode and pitch-catch regime; (**a**) 1.2 MHz 100 Vpeak excitation signal applied to a 1 nF capacitor; (**b**) The PSD of the AU response of a PWAS on an aluminum panel, in a pitch-catch regime, when excited at 500 kHz.

The first test case involved the measurement of drive excitation waveforms during Drive Monitor mode when applied to a 1 nF precision capacitor over the entire AUSAM^+^ bandwidth. The second test case involved measurement of responses in pitch-catch mode for a 6.3 mm diameter PWAS bonded to a 3 mm thick aluminum panel. Figure 13a shows a representative time history of the drive excitation for an optimized 1.2 MHz 5 cycle Hanning modulated tone-burst applied across the 1 nF precision capacitor, as measured by the AUSAM^+^ and the LeCroy and then further compared to the mathematical ideal signal. Figure 13b shows the Power Spectral Density (PSD) response of the 6.3 mm PWAS in a pitch-catch configuration when a 10 mm PWAS element is excited at 500 kHz. The results in both cases show a difference of less than 0.5% which does not increase when aggregated over the AUSAM^+^ bandwidth.

### 3.4. EM Impedance Performance 

**Figure 14:**
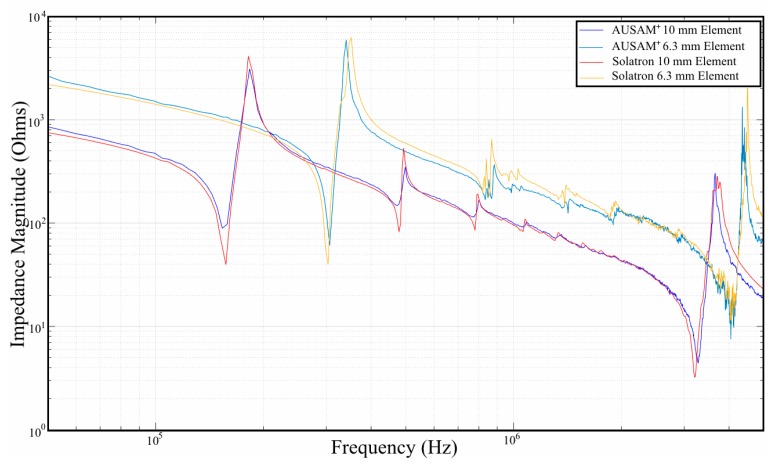
Comparison of the EM impedance magnitude sweep over the AUSAM^+^ bandwidth for un-bonded 6.3 mm and 10 mm diameter PWAS elements.

The AUSAM^+^ EM impedance measurement capability was assessed using a Solatron 1260 impedance analyzer as a reference instrument. To assess the performance, two free PWAS elements (10 mm and 6.3 mm diameter Pz27 discs) were analyzed using both systems over a frequency bandwidth of 50 kHz to 5 MHz. The results are shown in Figure 14. The AUSAM^+^ EM impedance magnitude plots are within 5% of the magnitude measured on the Solatron 1260. At the time of FOCT the phase component of the EM impedance was yet to be fully developed.

## 4. System Demonstrations

### 4.1. 2-D PWAS Wave Propagation Experiment

**Figure 15:**
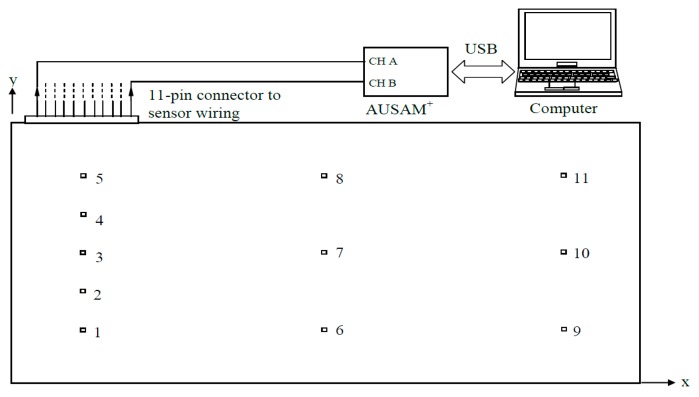
Schematic of experimental setup showing rectangular PWAS grid arrangement.

As a basic test of the Lamb wave excitation and reception capabilities of the AUSAM^+^ in pitch-catch and pulse-echo regimes, experiments were conducted on a 2024 aluminum alloy panel of 1.6 mm thickness. The panel was rectangular with side dimensions of 914 × 504 mm and was instrumented with a sparse array of eleven 7 mm square PWAS elements positioned on a rectangular grid. The locations of the PWAS elements on the panel are given in Table 2 relative to an origin at the bottom left corner of the panel, as shown in Figure 15.

**Figure 16:**
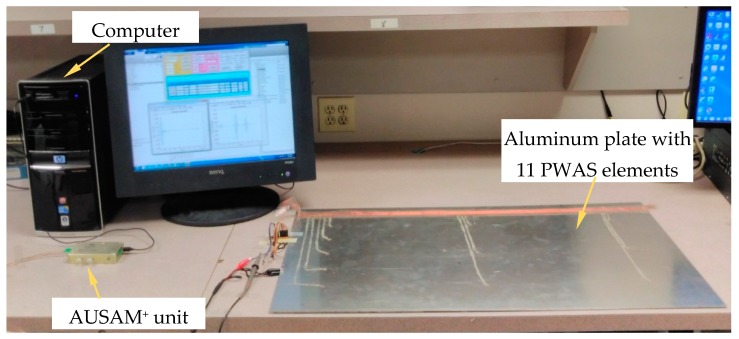
Image of the experimental setup.

PWAS 11 was used as the transmitter and all other PWAS elements were assigned as receivers. The excitation waveform applied to PWAS 11 was a Hanning-windowed six-cycle 300 kHz sinusoidal tone-burst with a 60 Vp-p amplitude. Figure 16 shows an image of the experimental setup.

#### Results

**Figure 17:**
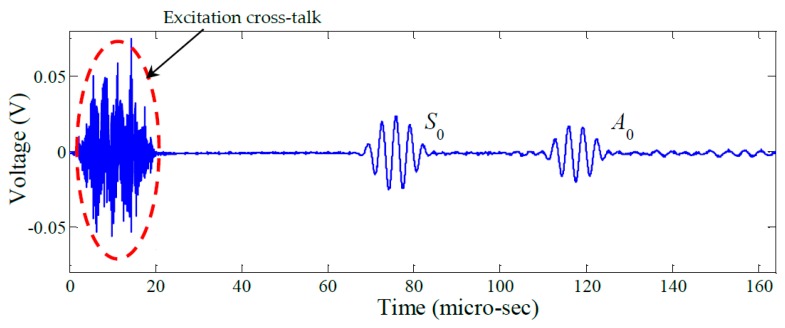
Typical signal acquired using the AUSAM^+^ (PWAS 8).

**Figure 18:**
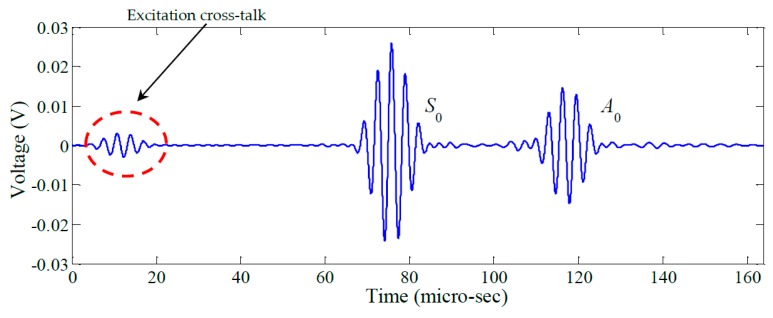
Signal band-pass filtered between 200 kHz and 400 kHz.

Figure 17 shows a representative signal acquired by one of the PWAS receivers during the experiment. It shows strong S_0_ and A_0_ wave packets and a relatively low background noise level. Figure 18 shows the same signal after applying a 200–400 kHz band-pass filter. This filter is seen to have noticeably affected only the excitation cross-talk.

**Figure 19:**
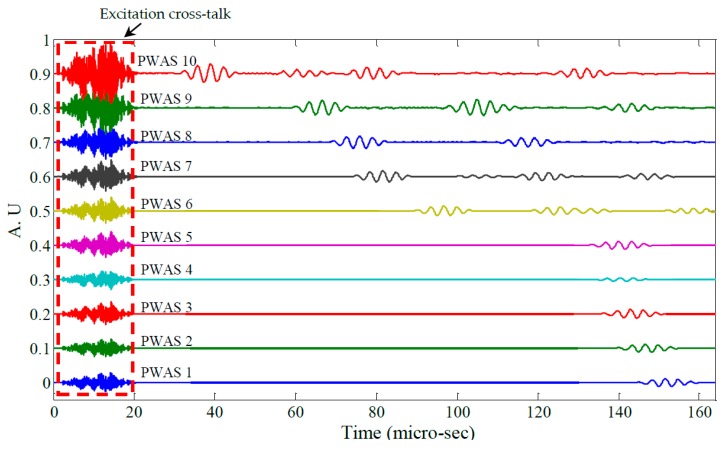
Raw signals from PWAS 1–10.

**Figure 20:**
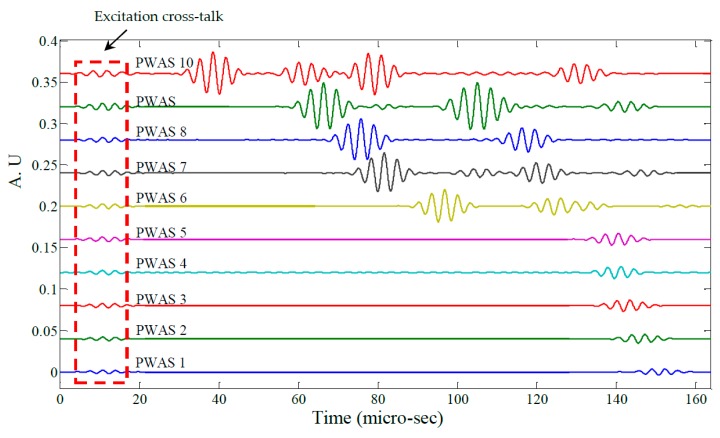
Filtered signals from PWAS 1–10.

**Figure 21:**
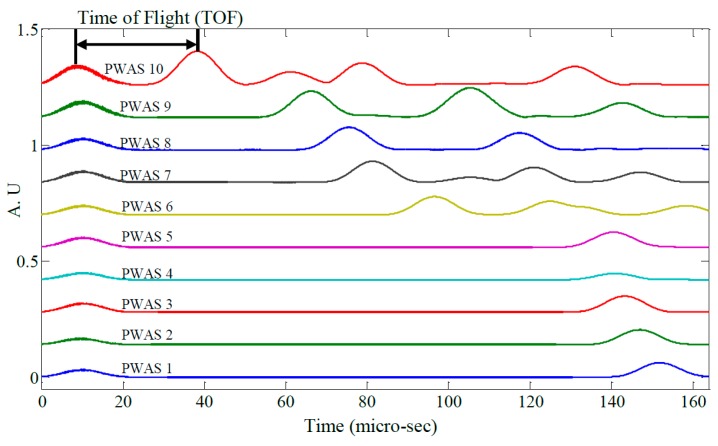
Signal envelopes of PWAS 1–10.

**Figure 22:**
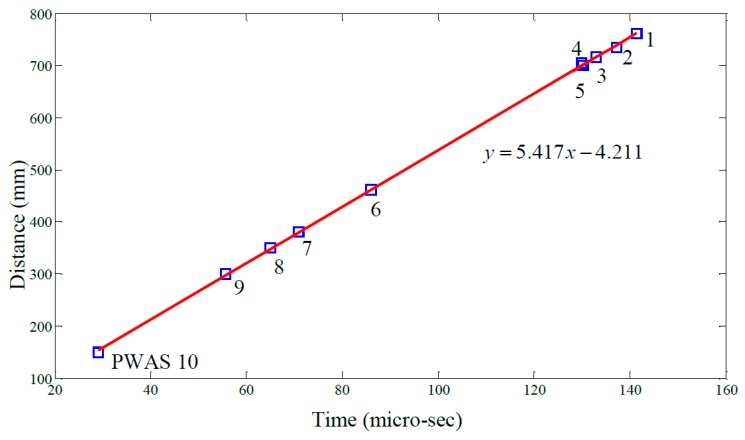
Correlation between radial distance and time of flight.

Figure 19 shows raw signals from the ten PWAS receiver elements and Figure 20 the same signals after band-pass filtering (200–400 kHz). Next, the signals were processed using a Continuous Wavelet Transform (CWT) producing the signal envelopes shown in Figure 21. The Time of Flight (TOF) for the S_0_ wave packet was determined for each PWAS and plotted as a function of radial distance from the transmitting PWAS shown in Figure 22. Linear regression was used to estimate the group velocity, yielding a value of 5.417 mm/μs which compares favorably to the theoretical group velocity for the S_0_ mode in the studied panel of 5.440 mm/μs.

**Figure 23:**
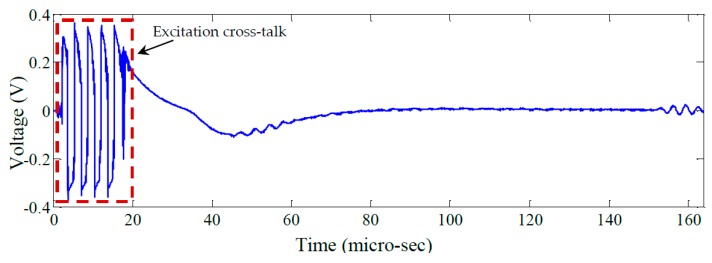
The unfiltered pulse-echo signal on PWAS 11.

**Figure 24:**
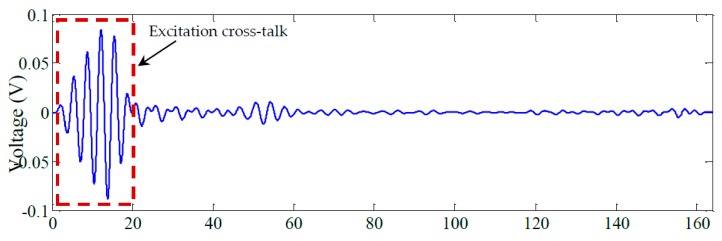
The filtered signal on PWAS 11.

Figure 23 shows the unfiltered pulse-echo response of PWAS 11 obtained using the AUSAM^+^ excitation signal configuration described previously. Figure 24 shows the same response after applying a band-pass filter (200–400 kHz).

The key issue here is the low-frequency distortion in the signal following the excitation burst which is a result of the TRS requiring time; approximately 80 μs; to reach steady state. Such distortion can interfere with acoustic signals returning within the recovery period as has occurred in this case with a return signal arriving approximately 45 μs after initiation of the pulse. This recovery period can be shortened by optimizing the excitation pulse to remove any low frequency transients and by utilizing the damping stage in the HVDA as mentioned previously.

### 4.2. Damage Detection—Aluminum Panel

The next test case considers a similar aluminum test panel but includes simulated structural damage in the form of a notch grown in depth and length over three steps with pulse-echo and pitch-catch interrogation applied between each step. As indicated previously, the AUSAM^+^ also has the ability to take EM impedance measurements to facilitate health checks on a transducer system. To demonstrate this, the experiment also incorporated damage to one of the PWAS elements in the form of de-bonding of the adhesive layer.

**Figure 25:**
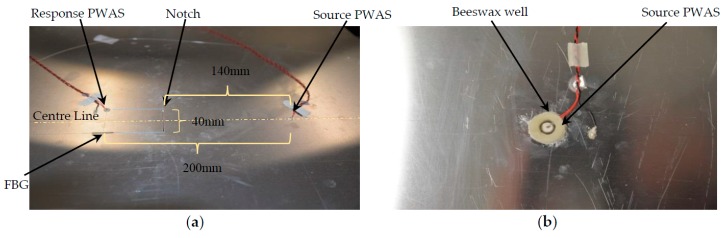
(**a**) An aluminum panel with 40 mm long full-depth extended notch located 140 mm from the source PWAS and perpendicular to the center line; (**b**) Photograph of the Beeswax well around the source PWAS to contain acetone solution used to induce damage of the bond layer.

The specimen was a 600 mm square 0.8 mm thick aluminum panel with two 6.5 mm diameter 0.5 mm thick PWAS discs bonded with silver-loaded epoxy and one 5 mm long FBG bonded using Noland UV cure adhesive, (see Figure 25a). The PWAS elements were placed equidistant from the panel center with a separation of 200 mm and connected to channels A and B of the AUSAM^+^, by a combination of twisted pair wire and MMCX coaxial cables. The positive wires were soldered atop the PWAS elements and the return ground wire connected to the panel, being circuit ground, using silver-loaded epoxy in close proximity to the respective PWAS. The FBG was positioned 40 mm from the sensing PWAS and equidistant from the source PWAS to minimize acoustic interference. The optical fiber was connected to a Yenista Tunics T100R Tunable Laser Source (Lannion, France) supported by two splitters, an optical circulator, and a Yenista CT400 Optical Component Tester (Lannion, France). The intensity of the output was interrogated using the AUSAM^+^ channel C PIN photodiode.

The response data presented for this test case corresponds to a single Hanning-windowed five-cycle sinusoidal drive pulse, except for channel C which recorded the PIN photodiode response of the FBG after 64 synchronous averages. The excitation frequency was swept from 100 kHz to 550 kHz in 50 kHz steps for both pulse-echo and pitch-catch regimes. Included at the beginning of each sweep was an EM impedance magnitude measurement taken by the module on each PWAS over a frequency range of 50 kHz to 5 MHz resulting in a total sweep time of approximately two minutes.

The peak excitation voltage and acquisition gain settings were selected to achieve a maximum response whilst ensuring no signal distortion or clipping occurred. The measured signals were processed using a windowed-sinc filter scaled by the excitation frequency $F_{exc}$ using the upper and lower cutoffs set equal to $F_{exc}+F_{exc}/2$ and $F_{exc}-F_{exc}/2$ respectively. A Hilbert transform was applied to aid the measurement of various mode arrival times according to the theoretical dispersion curves for the panel.

With no sensor array to gain spatial information for modal decomposition in the panel, and a sole reliance on response amplitude sensitivity for diagnostics, initial effort was directed to stability testing. Forty-eight sweeps were performed over multiple days to ensure repeatability of the baseline measurements before any damage was introduced to the panel or source PWAS.

A notch was carefully milled into the panel using a Dremel grinding disc N420 (2 mm thick) and grown in size over three steps. The notch location was 140 mm from the source PWAS and cut perpendicular to the panel center line, as shown in Figure 25a. The first cut was 20 mm in length and 0.4 mm in depth corresponding to the panel half thickness. The second cut increased the notch depth to penetrate the panel without changing its length. The third cut extended the length of the notch to 40 mm remaining equidistant about the center line and also at full panel penetration. Between each stage, ten sweeps were recorded for comparison to the baseline.

After simulating panel damage via the through-thickness 40 mm long notch, damage was introduced to the bond layer of the source PWAS by exposure to acetone. To maintain acetone saturation of the bond layer (i.e., to compensate for evaporation), the excitation PWAS was enclosed in a 5 mm high well of beeswax, (see Figure 25b). Since the beeswax attenuated the outgoing Lamb waves, a further ten sweeps were recorded before applying the acetone to establish a new baseline. With the beeswax well filled with acetone, sweeps were continually recorded over three hours.

#### Results

**Figure 26:**
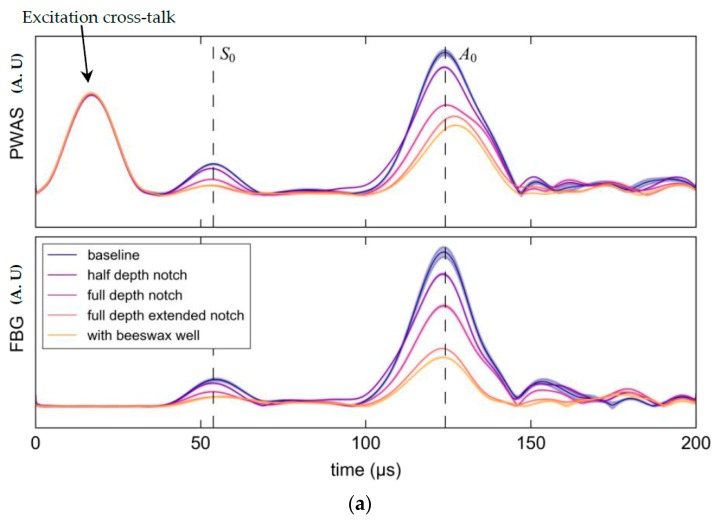

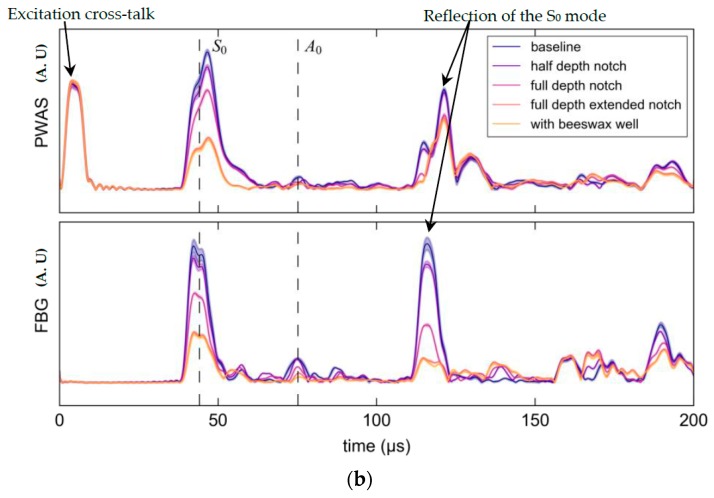
Hilbert transform envelops of the response PWAS and Fiber Bragg Gratings (FBG) showing the average and two standard deviations (shaded) for each stage in the experiment. (**a**) 150 kHz excitation; (**b**) 550 kHz excitation.

The initial repeatability test, i.e., the 48 sweeps over multiple days, showed good stability for impedance magnitude, pulse-echo and pitch-catch measurements at all frequencies for the PWAS elements. Stability was also good for the pulse-echo and pitch-catch measurements at all frequencies for the FBG. This is illustrated in Figure 26 by means of shading which represents two standard deviations about the mean response. All time histories in this experiment were similarly averaged over ten sweeps and presented in the same format, i.e., within a band of two standard deviations.

Two representative pitch-catch time histories are shown in Figure 26. The vertical dashed lines show the theoretical peak arrival time of the S_0_ and A_0_ modes, while the initial signal peak corresponds to excitation cross-talk. For the 150 kHz case in Figure 26a the A_0_ and S_0_ wave packets are easily distinguished with both showing a significant decline in amplitude with growth of the notch. Similar behavior was observed for the 550 kHz case however the A_0_ mode was barely excitable.

**Figure 27:**
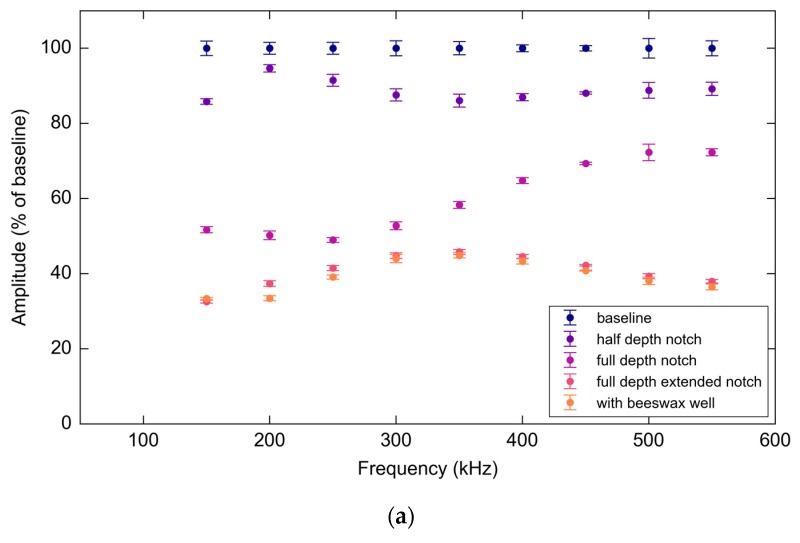

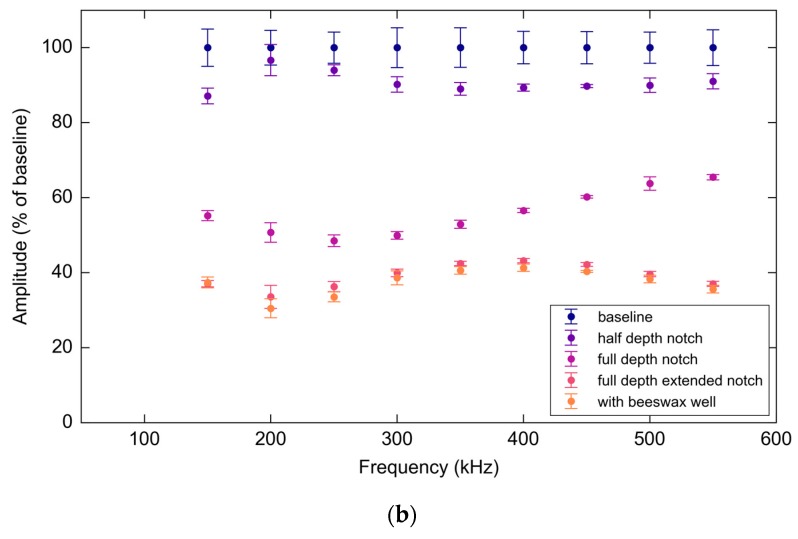
(**a**) PWAS S_0_ mode amplitudes normalized as a percentage of the baseline average peak over frequencies and experimental stages; (**b**) FBG S_0_ mode amplitudes normalized as a percentage of the baseline average peak over frequencies and experimental stages.

Figure 27 illustrates the trend in S_0_ mode attenuation for all test frequencies for both response PWAS and FBG. The S_0_ mode response PWAS and FBG data displays similar trends providing useful validation for both interrogation modalities.

**Figure 28:**
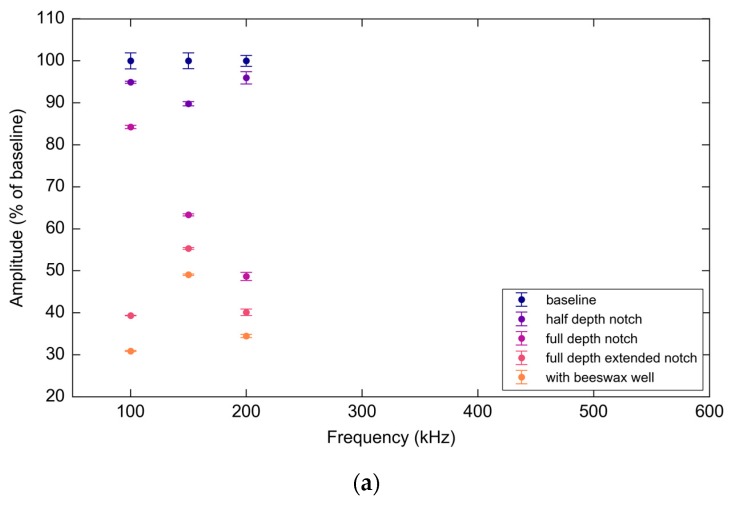

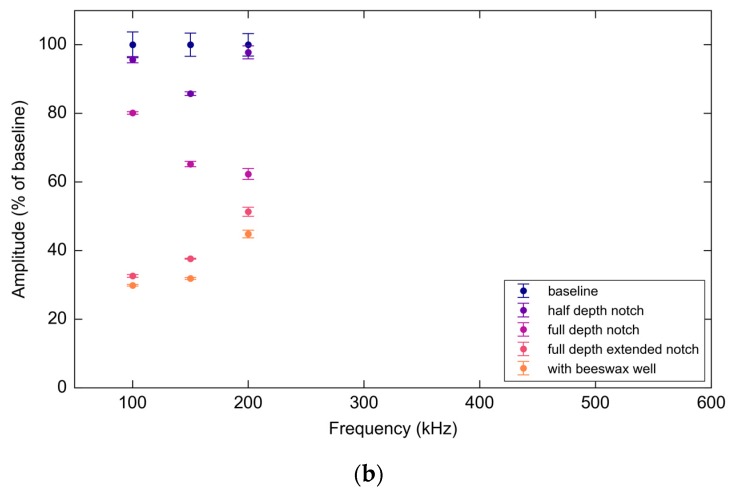
(**a**) PWAS A_0_ mode amplitudes normalized as a percentage of the baseline average peak over frequencies and experimental stages; (**b**) FBG A_0_ mode amplitudes normalized as a percentage of the baseline average peak over frequencies and experimental stages.

The amplitude of the A_0_ wave-packet was observed to decline rapidly with increasing frequency due to low excitability and was indistinguishable from the noise floor above 200 kHz in both the response PWAS and FBG measurements (see Figure 28). However, over this narrow bandwidth the A_0_ results still show a decline in signal amplitude with notch growth, though interestingly, by differing amounts for the two sensor types.

**Figure 29:**
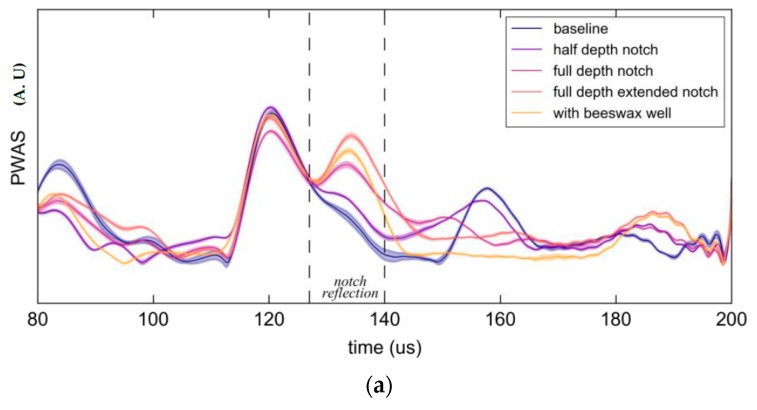

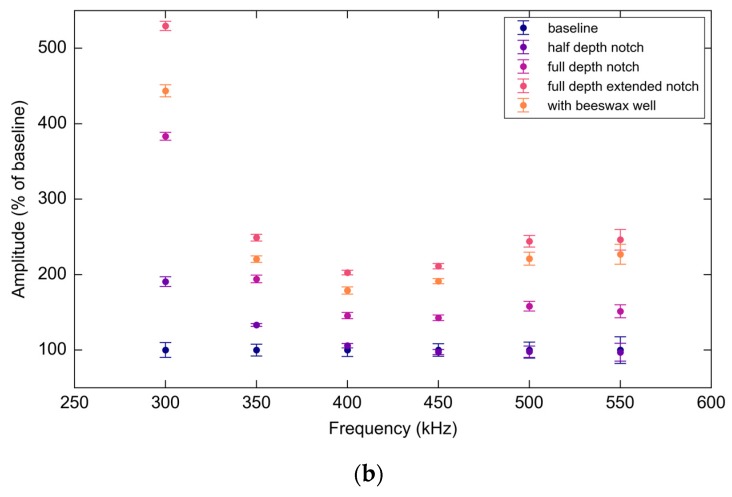
(**a**) Pulse-echo signal peak bordered by dashed lines corresponds to a reflection from the notch for each stage in the experiment with two standard deviations (shaded); (**b**) The same reflection Pulse-echo signal peak amplitudes normalized as a percentage of the baseline average peak over frequencies and experimental stages.

The pulse-echo data presented a more complicated picture but one broadly consistent with multiple reflection sources and mode conversion. The distortion caused by the settling time of the TRS was again evident but did not impact the following analysis due to the notch being sufficiently distant from the source PWAS. From the known Lamb wave velocities in the panel, the signal peak isolated and shown in Figure 29a, is surmised to be an A_0_ produced by mode-conversion of the incident S_0_ interacting with the notch. Figure 29b shows how the signal amplitude, expressed as a percentage of the baseline signal, varies as a function of panel state and excitation frequency. Error bars represent two standard deviations while some frequencies were omitted due to inconclusive data. In the last stage of the experiment shown in Figure 29b, the amplitude is observed to decline in response to the application of the beeswax around the source PWAS, as was expected.

**Figure 30:**
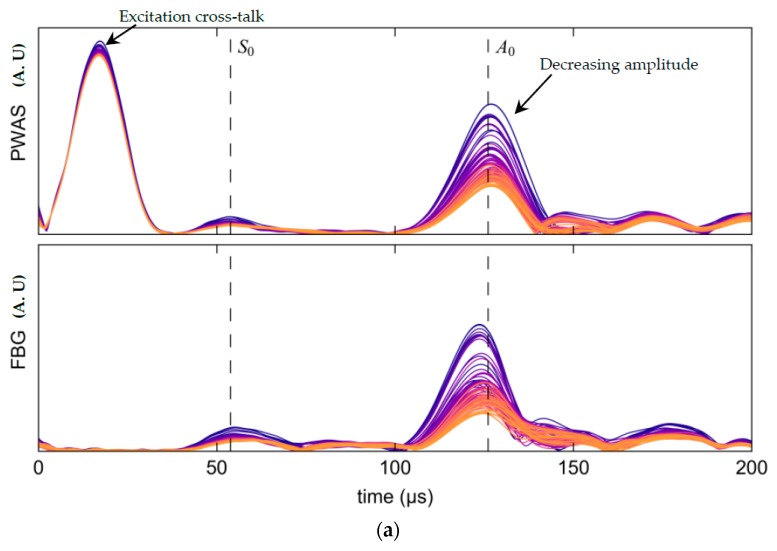

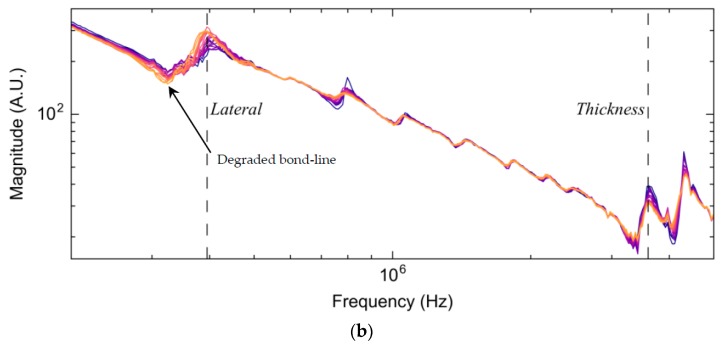
(**a**) Successive pitch-catch responses from dark (purple) to light (orange) at 150 kHz showing a gradual attenuation as the source element bond-line was damaged by acetone; (**b**) Successive impedance magnitude plots from dark (purple) to light (orange) showing an increase in capacitance at the lateral resonance peak indicating de-bonding of the source element.

Figure 30 illustrates the effect of de-bonding of the source PWAS element due to acetone exposure. A progressive decline in signal strength was recorded by both the PWAS and FBG sensors. However, the on-board EM impedance measurement allows a determination of the cause. Successive magnitude plots in Figure 30b show an increase in capacitance and lateral-mode resonant frequency which is consistent with a loss of stiffness in the bond-line. In other words, the change in sensor response in Figure 30a can be confidently attributed to a change in the acoustic source.

### 4.3. Aircraft Wing Skin Specimen

**Figure 31:**
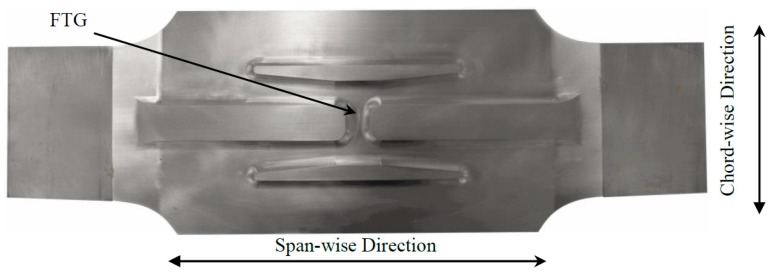
Test coupon representing key structural elements of F-111C lower wing skin at Forward Auxiliary Spar Station (FASS) 281.28. Marked is the location of the Fuel Transfer Groove (FTG).

Figure 31 shows a structurally detailed test coupon representative of the Forward Auxiliary Spar Station (FASS) 281.28 location in the F-111C lower wing skin. In Royal Australian Air Force (RAAF) F-111’s cracks were found to initiate at a stiffener depression, known as a fuel transfer groove (FTG), on the inside surface of the lower wing skin approximately mid span along the wing at FASS 281.28 [16].

The geometric complexity of the structure offers a more realistic test case than the simple panels considered in the previous two examples. There was no opportunity to grow a real crack in the coupon so a milled notch was introduced in the FTG at the approximate location of observed fleet cracking. The study was done using two PWAS elements interrogated in a pitch-catch arrangement connected to channel A and B of the AUSAM^+^ via a combination of twisted pair wire and MMCX coaxial cables.

**Figure 32:**
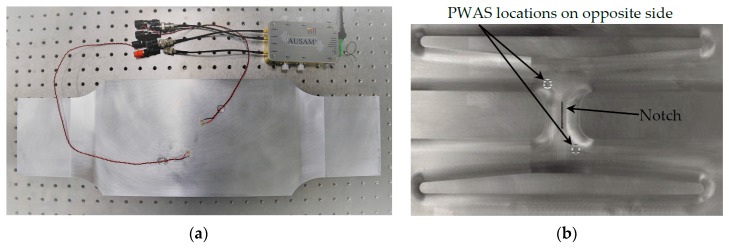
(**a**) The FASS coupon test set up showing two PWAS elements installed on the outside of the wing skin; (**b**) The notch milled into the FTG location after establishing a baseline to simulate where a crack typically forms under operational loading. Shaded marks show the location of PWAS elements bonded on the opposite side.

Two 5 mm diameter 1 mm thick PWAS discs were bonded to the external (clean) face of the wing skin coupon using silver loaded epoxy at an oblique orientation to the notch location as shown in Figure 32a. The attachment locations were determined from a previous acoustic wave-field survey using laser scanning vibrometry [17]. The pitch-catch response was measured across the frequency range 1 MHz–1.5 MHz in 50 kHz steps using a 5 cycle Hanning windowed tone-burst excitation signal. Synchronous averaging was not applied, so all time traces correspond to a single excitation pulse.

After establishing a baseline response for the pristine coupon, a notch was introduced. The notch was machined into the FTG resulting in a 20 mm long and 1.9 mm + −0.1 mm deep groove with its major axis parallel to the chord-wise direction (see Figure 32b). The excitation and acquisition gains were optimized to ensure a clean signal with no distortion or clipping.

#### Results

**Figure 33:**
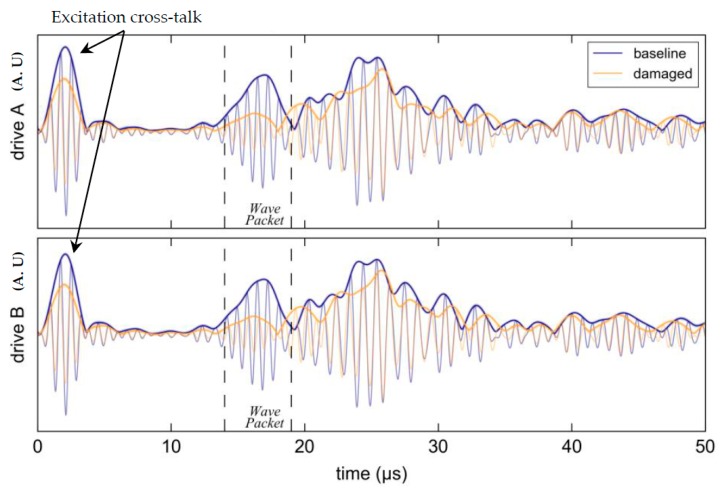
Effect of the notch on the pitch-catch response at 1.25 MHz with the first incident wave-packet bordered by dashed lines. The barely perceptible shading confirms very good repeatability.

All response data were processed using a windowed-sinc filter in the manner of Section 4.2. Figure 33 shows the response for the mid-band excitation frequency of 1.25 MHz with the top plot showing the response from an applied excitation signal to PWAS channel A and the bottom plot for an excitation signal applied to PWAS channel B. Changing the direction of the incident wave field had little effect on the response, as expected given the symmetrical PWAS placement and coupon geometry. The Hilbert transform envelope is from an average of ten measurements with 2 standard deviations of variation shown by shading.

The attenuation in the first wave-packet caused by the notch was both strong and relatively consistent across the selected band, confirming that the chosen interrogation regime offers a potentially good basis for inspecting this particular structure. The variation in cross-talk amplitude is a result of variation in placement of the unshielded twisted pair PWAS connections and has no bearing on the acoustic signal.

**Figure 34:**
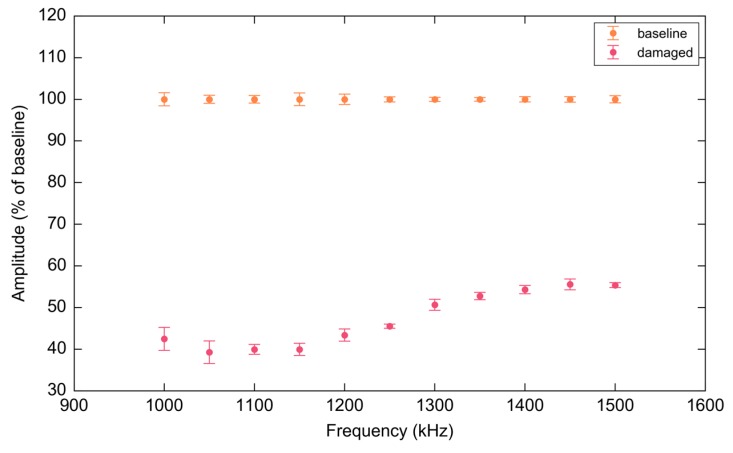
Variation in attenuation expressed as a percentage of the baseline calculated using a Riemann sum of the signal envelope for the first wave-packet over the experimental frequency range. Error bars represent two standard deviations over ten consecutive sweeps.

Figure 34 illustrates the effect of the notch on the strength of the first wave-packet as a function of excitation frequency. The largest deviation in response occurs at 1.1 MHz which is clearly an optimal frequency for inspection within the considered band.

### 4.4. Composite Aerospace Specimen

Whilst composite materials are well suited to light weight construction they have much lower tolerance to mechanical impact compared to metallic structures and can suffer significant damage from even relatively low velocity impacts [18]. This has driven considerable interest in the development of more efficient methods for impact damage detection including structural health monitoring using embedded PWAS elements. The final test case considers such a scenario.

Examined is a composite-honeycomb sandwich coupon consisting of 15 mm thick Nomex^®^ honeycomb sandwiched between two composite face sheets each approximately 0.7 mm thick. During manufacture of the coupon one of the laminate skins was instrumented with two PWAS elements bonded to the inside surface 140 mm apart. Small sections of honeycomb core were excised to accommodate the elements and to allow egress of the wires from the side of the specimen. The face sheets and core were then assembled and film adhesive used to create the final sandwich structure.

**Figure 35:**
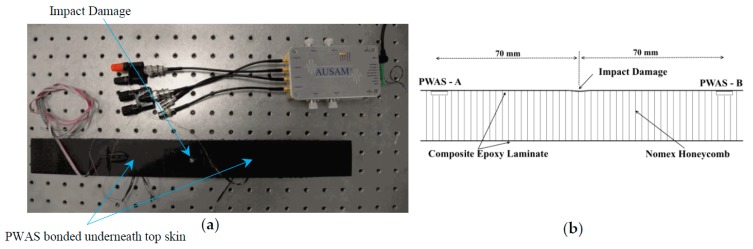
Composite test set-up. (**a**) Interrogating the impacted coupon; (**b**) coupon cross-sectional view showing the location of two embedded PWAS elements in relation to the impact site.

After establishing a baseline response for the coupon an impact was applied at the center of the instrumented face sheet using a 4.623 kg mass with 12.7 mm diameter hemispherical impact. The drop height was 148 mm corresponding to energy of 6.7 J. Figure 35a shows the impact damaged coupon and Figure 35b a schematic cross-sectional view of the structure which includes the distance between the PWAS elements and the impact location.

The embedded PWAS were connected to channel A and B of the AUSAM^+^ via a combination of twisted pair wire and MMCX coaxial cables. After impact damage was applied, the coupon responses in the pitch-catch regime were compared to the baseline in both directions at frequencies between 250 kHz and 400 kHz, swept in 50 kHz steps. A prior survey of frequencies over the entire AUSAM^+^ bandwidth showed no measureable response above 400 kHz most probably as a result of material attenuation.

#### Results

**Figure 36:**
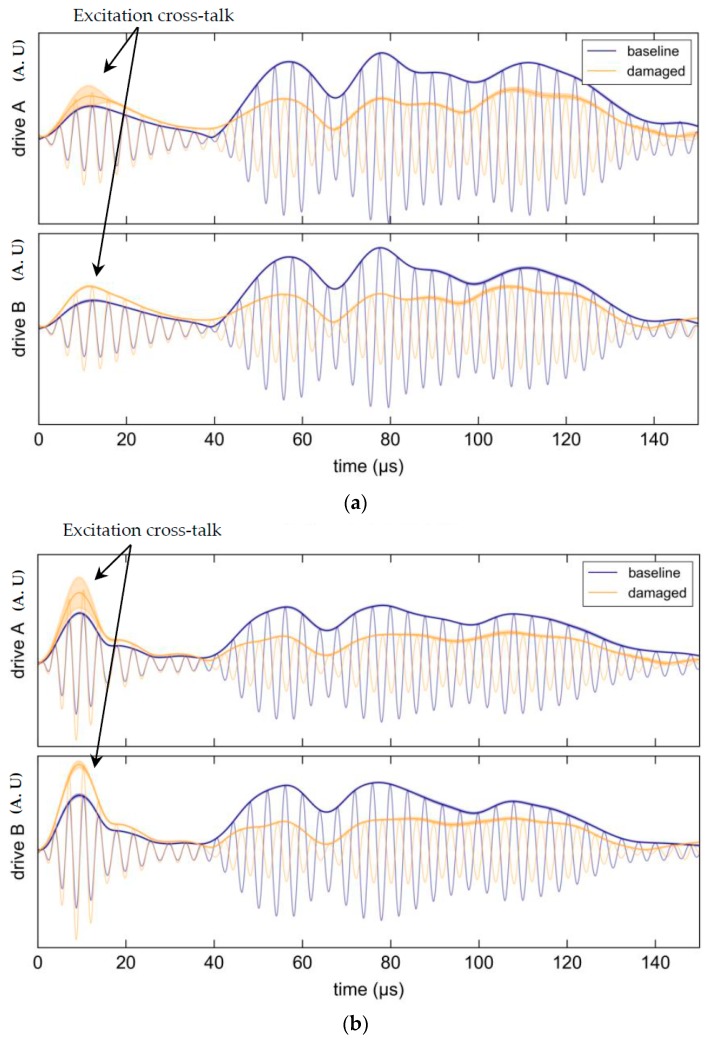

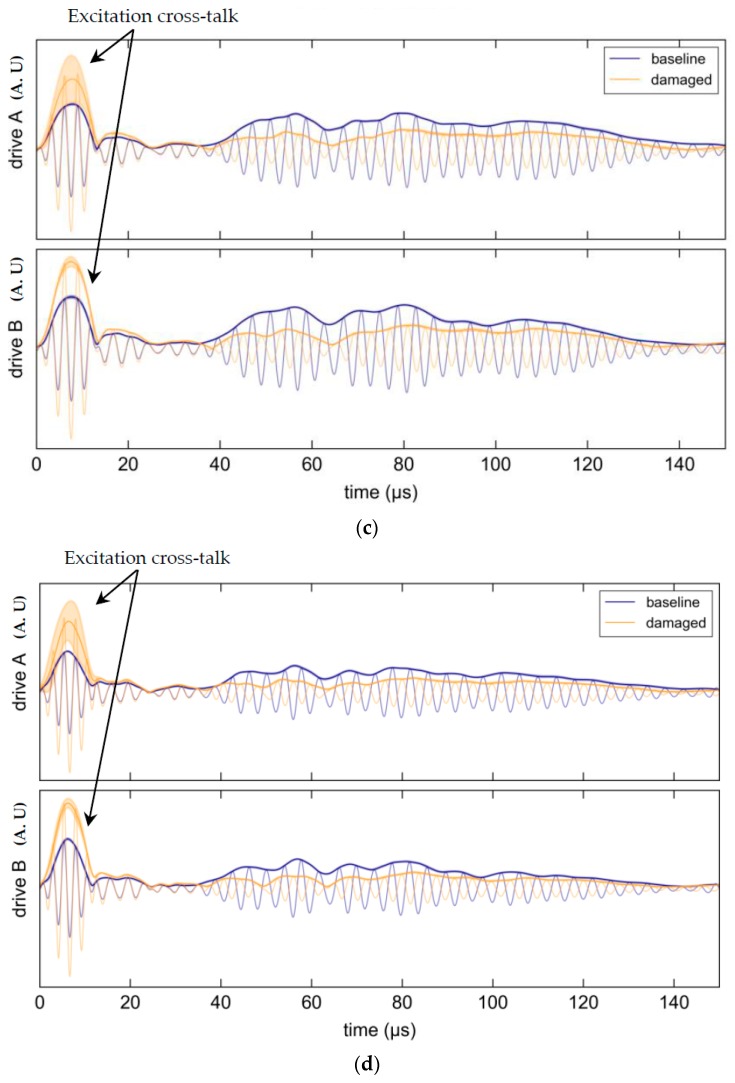
Waveforms for pitch-catch interrogation in both directions; top plots show excitation on PWAS channel A and bottom plot shows excitation on PWAS channel B. The Hilbert transform envelopes are displayed and any shading represents two standard deviations in variability over 10 consecutive sweeps: (**a**) 250 KHz; (**b**) 300 KHz; (**c**) 350 KHz; (**d**) 400 KHz.

**Figure 37:**
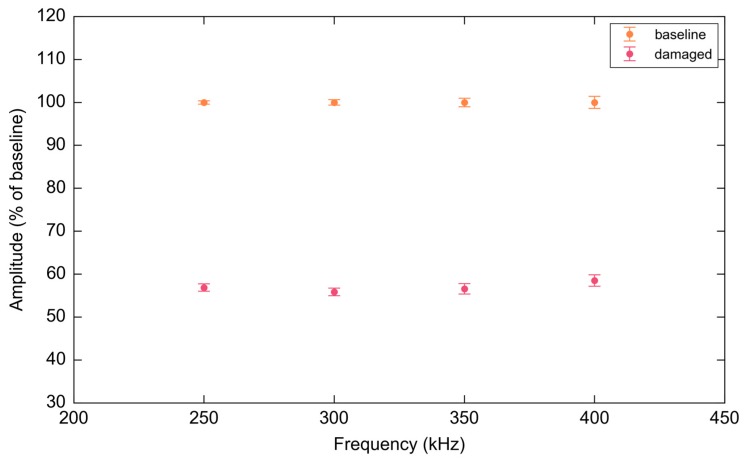
Attenuation due to coupon damage as a normalized percentage of the baseline calculated using a Riemann sum of the signal envelope in the time window 40–150 μs. Error bars represent two standard deviations showing the variability over ten consecutive sweeps.

As before, all waveform measurements were processed and repeated ten times. Figure 36 shows the mean envelope (solid line) and two standard deviations (shaded) of the sampled waveforms for each frequency. Strong attenuation is seen over the considered time window for all frequencies and is largely independent of the direction of the incident wave field. The consistency of attenuation across the considered frequency range is illustrated in Figure 37.

The impact damage applied in this case was beyond the threshold of barely visible impact damage and thus relatively severe. Further work is planned to assess the performance of the AUSAM^+^ under more moderate levels of impact damage.

## 5. Discussion and Future Work

Although the performance tests described in Section 3 and Section 4 were not exhaustive the results nonetheless offer a useful demonstration of key capabilities and generally affirm that most of the design goals set out for the device were achieved. From the viewpoint of facilitating aerospace deployment of embedded SHM systems it is particularly significant that the results were achieved with a device that is compact and lightweight, manages power efficiently, is robust to EMI, has a capacity to handle both PWAS and optical FBG sensors and can operate synchronously with other units in networked installations.

However, the work has also highlighted room for improvements in HVDA excitation quality which untreated can contain residual low frequency distortion that increases the TRS recovery time. As previously mentioned, this impacts the pulse-echo interrogation regime by masking information from structural defects in close proximity to the actuated PWAS. A remedy for this can be found in software improvements alone and does not necessarily reflect a limitation of the hardware. Employing advanced iterative optimization algorithms during HVDA waveform generation, and or including a more realistic HVDA switch model when generating the excitation first attempt is presently being developed.

Despite the work performed to characterize the acquisition system in Drive Monitor mode, aspects of the EM impedance capability are still not mature. Jitter in the ADC acquisition window spanning one sample clock period has increased the time required to produce phase information while unmeasured AFE characteristics still impact EM impedance accuracy. Again, it is believed these limitations can be addressed by software improvements alone.

**Figure 38:**
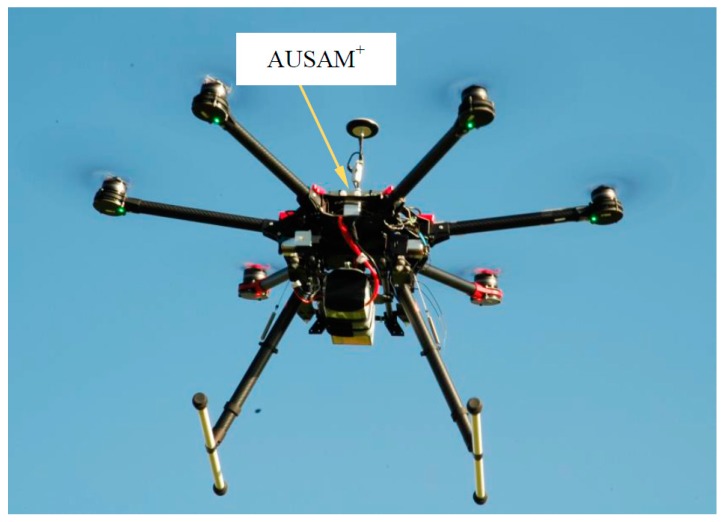
AUSAM^+^ strapped on-board a SJ900 Hexacopter during flight testing.

Recent work at the Defence Science and Technology Group (DSTG) has employed the AUSAM^+^ controlled by a Raspberry Pie Zero SOC to interrogate the cylindrical composite arm of a SJ900 hexacopter drone under real-time flight loading, (see Figure 38). This setup goes some way to demonstrate the flexibility of the AUSAM^+^ by virtue of its low mass, size, Wi-Fi enabled interface and ease of use to adapt quickly to various sensor bed platforms.

As mentioned in Section 2.5, upgrading the FPGA with one that includes an on-board ARM core processor is already underway and effectively allows the Wi-Fi or 3G capability tested on the drone to be added by simply replacing the commercial FPGA daughter board. The Linux OS installed on such a processor will allow full autonomous control of the AUSAM^+^ hardware object and can provide real time on-board data analysis opening the way for non-cyclic structural interrogation regimes. Such future work will relax the USB 2.0 power constraint enabling the acquisition system to run continuously while the on-board processor can apply algorithms to monitor, log and possibly characterize in real time passive acoustic emission events in aerospace structures.

## 6. Conclusions

The AUSAM^+^ provides an example of robust, purpose built and simple to use test equipment employed for on-demand damage detection required for condition-based maintenance approaches on aerospace structures. This highly flexible system allows custom interfaces to suit the challenges of embedding PWAS sensors and integrating next generation optical AU interrogation modalities such as FBGs into high value airframe structures due to its size, low mass, ruggedized packaging and intuitive hardware object. The on-board EM impedance capability supports the requirement for realistic embedded SHM systems to self-diagnose element degradation thereby reducing false positives.

The AUSAM^+^ bandwidth opens the way to exploit higher order Lamb wave inspection regimes, while the novel HVDA provides the drive flexibility and authority to target these modes. These case studies demonstrated the ease with which one can utilize the AUSAM^+^ Matlab or Python hardware object to quickly set up custom SHM regimes and apply them to various aerospace structures. The AUSAM^+^ system not only offers a flexible laboratory tool to investigate the many scientific and engineering challenges associated with embedded SHM research but could also provide a robust hardware solution for operational and potentially in-flight applications.

## Figures and Tables

**Table 1:** AUSAM^+^ acquisition system AFE channel configurations when Drive Monitoring is selected.

HVDA Excitation Channel	Voltage Measurement Channel	Current Measurement Channel	Configuration State (All PIN Photodiode Opt Buffer Amplifiers = Disabled)
Channel A or Channel B	Channel D	Channel C	Channel D Voltage Monitor switch = ON Channel D TRS = OFF Channel C TRS = OFF Channel C Current Amplifier = Enabled Channel B Current Amplifier = Disabled
Channel C or Channel D	Channel A	Channel B	Channel A Voltage Monitor switch = ON Channel A TRS = OFF Channel B TRS = OFF Channel B Current Amplifier = Enabled Channel C Current Amplifier = Disabled

**Table 2:** Location of PWAS elements on rectangular panel.

PWAS	1	2	3	4	5	6	7	8	9	10	11
x (mm)	100	100	100	100	100	450	450	450	800	800	800
y (mm)	100	175	250	325	400	100	250	400	100	250	400
